# Social Norms Change and Tobacco Use: A Protocol for a Systematic Review and Meta-Analysis of Interventions

**DOI:** 10.3390/ijerph182212186

**Published:** 2021-11-20

**Authors:** Shaon Lahiri, Jeffrey B. Bingenheimer, William Douglas Evans, Yan Wang, Priyanka Dubey, Bobbi Snowden

**Affiliations:** 1 Department of Prevention and Community Health, Milken Institute School of Public Health, The George Washington University, 950 New Hampshire Ave. NW, Washington, DC 20052, USA; bartbing@gwu.edu (J.B.B.); wdevans@gwu.edu (W.D.E.); yanwang20@gwu.edu (Y.W.); 2 Department of Community and Behavioral Health, University of Iowa College of Public Health, 145 N Riverside Dr., Iowa City, IA 52242, USA; priyanka-dubey@uiowa.edu; 3 Department of Global Health, Milken Institute School of Public Health, The George Washington University, 950 New Hampshire Ave. NW, Washington, DC 20052, USA; bsnowden01@gwu.edu

**Keywords:** social norms change, tobacco use, meta-analysis

## Abstract

Tobacco use kills more than eight million individuals each year, and results in substantial economic and human capital loss across nations. While effective supply-side solutions to tobacco control exist, these approaches are less effective at promoting cessation among heavy smokers, and less feasible to implement in countries with weaker tobacco control policy environments. Thus, effective demand-side solutions are needed. Shifting social norms around tobacco use is one such promising approach. To this end, a systematic review and meta-analysis of social norms intervention studies to influence tobacco use will be conducted following PRISMA 2020 guidance. Tobacco intervention studies with at least two time points that explicitly mention social norms or social influence as part of an intervention or set of measured variables will be included. Literature sources will comprise PubMed, Scopus, PsycInfo, and the Cochrane Trial Registry, as well as several grey literature sources. Two reviewers will independently screen studies, and risk of bias will be assessed using the Cochrane Risk of Bias 2 and ROBINS-I tools. The primary outcomes will be change in tobacco use and change in social norms. A random-effects meta-analysis will be conducted for both outcomes. Sources of heterogeneity will be explored using meta-regression with key covariates. Non-reporting biases will be explored using funnel plots. PROSPERO: CRD42021251535.

## 1. Introduction

### 1.1. Tobacco Use and Control

Tobacco use is pernicious. It kills more than eight million individuals per year, and is practiced by approximately 1.3 billion users, over 80% of whom reside in low- and middle-income countries [1]. Estimates from 2018 suggest that 82% of smokers are male, and 1.2 million non-smokers are exposed to second-hand smoke [2]. Tobacco-related illnesses include eight of the leading causes for premature mortality, including ischemic heart disease, cerebrovascular disease, chronic obstructive pulmonary disease, tuberculosis, lower respiratory infections, and cancers of the trachea, bronchi, and lungs [2]. The economic costs of tobacco-related illnesses are also substantial, and typically include direct healthcare costs and indirect costs related to lost productivity arising from tobacco-related morbidity and mortality. An estimate of the total economic costs related to tobacco smoking in 2012 was $1.436 trillion, with nearly 40% of this figure occurring in developing countries [3]. However, this estimate does not include all countries where tobacco smoking is prevalent, (The United Nations currently recognizes 193 Member States [4]. The estimate of total economic costs of tobacco smoking was based on 152 countries, and is therefore an underestimate of the true global economic costs of tobacco smoking.) nor does it include smokeless tobacco-related economic costs. Global economic estimates of smokeless tobacco are not currently available. However, the total economic costs related to smokeless tobacco use for 2017–2018 in India, which represents two-thirds of the global population of smokeless tobacco users, were estimated to be approximately $27.5 billion [5]. In this way, tobacco use and related illnesses represent a global epidemic, depleting the human capital and economic potential of nations.

A number of strategies have been used to prevent initiation and promote cessation of tobacco use. Supply-side measures typically leverage state or institutional authority to change the availability, affordability, and acceptability of tobacco products. These measures include modifications to the supply chain such as tobacco crop diversification, restrictions on smoking in public places and schools, restricting tobacco sales to minors, and including salient health warnings on tobacco products [6]. Some of the most effective approaches for reducing tobacco use involve the implementation of specific excise taxes (i.e., based on quantity or weight) or ad valorem excise taxes (i.e., based on a percentage of the value of the product) which increase the retail price of tobacco [7,8,9,10]. While excise taxes have been found to delay onset of smoking, they appear to be less effective at promoting cessation among existing smokers, and particularly among heavy smokers [11,12,13]. Evidence for equity impacts (i.e., whether an intervention has a greater impact on individuals of lower or higher socioeconomic groups) of supply-side interventions is also mixed [14]. Additionally, the tobacco industry is attracted to many low- and middle-income countries where the tobacco control policy environment is weaker, and has expanded to such markets elevating smoking prevalence [15]. Thus, while supply-side interventions are effective for many across diverse populations, they are less effective for hard-to-reach populations and in settings with limited institutional tobacco control capacity.

By complement, many approaches have also been used to reduce the demand for tobacco products. These include mass media campaigns, the creation of smoke-free environments, tobacco cessation services, and the adoption of cessation aids [13]. Some effective demand-side approaches include group behavioral therapy, Nicotine Replacement Therapy (NRT) and medication, and counselling interventions [16]. It should be noted however, that many of these approaches are based on controlled clinical efficacy studies rather than real-world population-level effectiveness studies. Existing evidence of non-price policies and interventions on tobacco use is mixed, and there is substantial heterogeneity in implementation, cessation effects, and equity impacts [13,14,17,18]. The WHO Framework Convention on Tobacco Control (WHO FCTC), one of the most widely endorsed treaties in the world, specifies various tobacco control measures for signatories, including price interventions, smoke-free policies, and tobacco advertising bans [19]. However, the FCTC does not specify specific “demand reduction” measures beyond advocating for the provision of counseling services (Article 14). Thus, evidence for effective demand-side interventions is sorely needed.

### 1.2. Social Norms and Tobacco Use

Related to demand-side factors influencing tobacco products, prevailing social norms around tobacco use have been suggested to form through social exposure [20]. Social norms typically refer to perceptions of the prevalence of behavioral enactment (“descriptive norms”), and perceptions of the approval or disapproval of a behavior by important others (“injunctive norms”) [21]. Studies of social norms also explore anticipated or actual rewards for norm deviation and compliance [22]. Social norms are distinct from other collective behavioral phenomena such as habits, customs, and moral rules because these are not conditional on perceptions of others, whereas social norms are conditional on perceptions of others’ behavior and approval [22]. Exposure to pro-smoking social norms is associated with individual tobacco use, especially during adolescence and young adulthood when tobacco use typically begins [23,24]. Longitudinal evidence suggests that smoking initiation is associated with pro-smoking social norms [25]. In particular, evidence from multiple studies in different countries suggests that observing others’ tobacco use, and having friends who use tobacco, are significantly associated with one’s own tobacco use [26,27,28]. While a peer group can be a strong source of normative influence on individual tobacco use, parental smoking can be influential as well, and the collective smoking prevalence in a social environment may even influence children whose parents do not smoke [26]. Normative influence is not just limited to direct observation of others. It can also manifest in media exposure. While the tobacco industry has long capitalized on this effect pathway, more recent evidence suggests that pro-tobacco depictions in media can act as a “super peer” in influencing adolescent social normative perceptions, suggesting an even more potent role of pro-tobacco media [29].

Beyond perceptions of how common and dis/approved smoking is within a milieu, social norms around smoking also manifest as stigma against smokers. For example, smoking stigma in the United States (US) is suggested to have formed as a result of workplace discrimination policies in hiring smokers, fears of second-hand smoke harming children, lower levels of education, and disapproval of smoking by family and friends [30]. Stigma can be internalized, manifesting as lower self-efficacy and motivation to quit smoking, avoidance of treatment-seeking, or defensive coping consequences such as increased self-esteem of smoking as a result of public backlash against the practice [31]. Stigmatizing smokers may also worsen health outcomes for already vulnerable individuals, such as ethnic/racial minorities, sexual or gender minorities, individuals with mental illness, and those of lower socioeconomic status (SES). In particular, the relationship between SES and internalized stigma tends to reflect the social gradient of smoking within society [32,33].

The risks of stigma appear to be multiplicative at the intersection of multiple already stigmatized identities. For example, Lipperman-Kreda et al. [34] found that low-income African American sexual and gender minorities in the US experienced greater smoking-related stigma compared with other sexual and gender minorities. The intersection of African-American race with sexual and gender minority status in this context resulted in greater experienced stigmatization, among an already vulnerable low-income community. Similarly, among a sample of Australian women, Triandafilidis et al. [35] found that women with multiple cultural, gender, and class identities faced compounding stigma due to smoking. In this way, punitive social norms around tobacco use can manifest in stigma against smokers broadly, but also against specific demographic or socioeconomic groups.

Social norms may be a valuable entrypoint for tobacco control efforts, based on longitudinal social network evidence suggesting that cessation behavior can spread in a network through proximal and distal ties [36,37,38]. Social norms interventions for tobacco use often involve correcting normative misperceptions (e.g., [39]) and using peer leaders to drive activities and dialogue around change (e.g., [40]). The evidence base for these approaches is mixed, and further research is needed to understanding if and how social norms add value to tobacco reduction, cessation, or prevention interventions. Synthesizing this literature is challenging because the social norms scholarship is extremely diverse, reflecting decades of scholarship from multiple disciplines, and based on different conceptualizations of normative constructs.

One approach to synthesizing the literature on interventions that leverage social norms or social influence to shift tobacco use is to focus on the “active ingredients” or underlying mechanisms that are most effective [41], and in which settings they tend to function best. Understanding the conditions under which social norms interventions do not work is just as important as understanding the conditions under which certain interventions do work. Therefore, this review aims to synthesize the literature on social norms interventions, defined as studies in which social norms or social influence changes are measured as an outcome, predictor, or mediator, or in which social norms or social influence are an explicit part of an intervention even if they are unmeasured. In such cases, a normative or social influence framework might be used to guide an intervention (e.g., leveraging influential peer leaders to disseminate normative messages, employing peer resistance skills to counter social normative pressure, etc), though the measured outcomes may not include social norms, and only tobacco use (see for example Telch et al. [42] which uses a social influence approach but does not measure normative changes). Five mechanisms underlying social norms interventions have been proposed by Legros & Cislaghi [43] based on a review of reviews of the theoretical literature:

1. Correcting misperceptions
2. Structural changes
3. Legal reforms
4. Role models
5. Power dynamics

These proposed mechanisms are a useful starting point for this review, and more mechanisms may yet be found to underlie social norms interventions.

### 1.3. Research Questions and Scope of Review

This review focuses on four key dimensions of social norms interventions for tobacco use, which are highly relevant to policy and programs: effectiveness, measurement, mechanism, and modality. In this regard, the aim is to determine to what degree are social norms interventions effective at influencing both social norms around tobacco use, and tobacco use itself.

These aims give rise to the following research questions which will be addressed by this review:

1. Are interventions that aim to influence social norms around tobacco use effective? If so, how effective are they, and what intervention modalities are most effective? Which intervention modalities appear to be less effective at tobacco use change, and under what conditions?
2. Among interventions that attempt to influence tobacco use, how are social norms conceptualized and measured?
3. Among interventions to influence tobacco use that involve social norms, what social norms change mechanisms are most commonly used?

## 2. Materials and Methods

This protocol follows the PRISMA-P checklist for systematic review protocols [44]. A completed PRISMA-P checklist is available in Supplementary File S1.

### 2.1. Eligibility Criteria

#### 2.1.1. Population

The eligible population for this study is human subjects.

#### 2.1.2. Interventions

This review focuses on social norms intervention studies, defined as an intervention study in which social norms or social influence are measured as an outcome, predictor, or mediator, or have an explicit role in the intervention or policy being evaluated but are unmeasured. This is intended to be deliberately broad as many different intervention modalities exist for influencing social norms, and a broad criterion such as this is intended to capture them in order to determine their underlying change mechanisms. These interventions may also be events, such as the passage of a law or policy. Studies without an explicit intervention or event being evaluated will be excluded. This includes longitudinal prospective studies of development trajectories that do not evaluate a specific intervention or policy. Additionally, ’intervention’ is defined as “coordinated sets of activities designed to change specified behaviour patterns” [45].

#### 2.1.3. Comparators

Quasi-experimental and experimental studies will be included in this review to capture a broad range of intervention studies. For experimental studies, eligible comparators are no treatment, treatment-as-usual, or an alternate treatment. For quasi-experimental studies, no comparator is necessary. Given that social norms interventions may not involve comparators due to ethical or logistical challenges, a broad comparator criterion was deemed appropriate.

#### 2.1.4. Outcomes

This review will focus on two broad outcome groups—those related to tobacco use, cessation, prevention or uptake, and those related to change in social norms. Eligible tobacco outcomes will include all “tobacco products”, based on the definitions in the World Health Organization’s Basic Handbook on Tobacco Product Regulation [46]. These include traditional combustible (e.g., cigarettes) and non-combustible products (e.g., smokeless tobacco), as well as newer electronic products (e.g., electronic nicotine delivery systems, vapes, Juuls) and heated tobacco products (e.g., IQOS). Outcomes related to medication or cessation products and services will also be eligible, as the focus is on the reduction of tobacco and related products. Both self-reported and biometric (e.g., salivary measures) tobacco outcomes will be included. Outcomes related to an anticipated tobacco or cessation product, such as market research studies ahead of product launches, will not be included.

Given the many conceptualizations of social norms, this review will focus on changes in perceptions related to the perceived frequency or acceptability of tobacco use, within a specific social group (e.g., friends, society, etc). Outcomes related to perceived reward or sanctions as a result of norm compliance or deviation will be acceptable as well. Indeed, any outcome with the term ’norm’ will be included, as social norms may at times be called peer, gender, cultural, or other norms. Additionally, any studies with measurable social influence effects will be included as well, given that social norms are sometimes understood as social influence in the tobacco use literature. Studies that do not quantitatively assess a social norms construct in the manner described, but do explicitly describe normative or social influence intervention components will be included.

#### 2.1.5. Study Designs

Eligible study designs will have tobacco and social norms measurements for at least two time points. Experimental and quasi-experimental intervention studies will be eligible. Experimental studies include randomized controlled trials or controlled clinical trials. Quasi-experimental studies include one group pre-post, regression discontinuity, time series, difference-in-difference, and instrumental variable regression designs. One time cross-sectional studies will not be eligible. Mixed methods studies will be included if they include a quantitative component matching inclusion criteria, and purely qualitative studies will not be eligible. All types of reviews (e.g., systematic, rapid, scoping, etc.) will be excluded, but their references may be checked for quantitative studies matching inclusion criteria.

There is no time restriction for this review, and only studies published in English will be included. Studies published in peer-reviewed journals, as well as gray literature will be included.

### 2.2. Search Strategy and Information Sources

A search strategy was developed based on pilot search results and in collaboration with a systematic review librarian at George Washington University. The search strategy will be used in the following peer-reviewed literature databases: Scopus, PubMed, PsycInfo, and the Cochrane Trial Registry. It will also be applied in the following gray literature sources: Society for Research on Nicotine and Tobacco (SRNT), MedRxiv, Clinicaltrials.gov, Open Science Framework, Proquest Dissertations, and the website of the Truth Initiative. The complete search terms for PubMed is available in Supplementary File S2.

The search terms related to tobacco and social norms were informed by a review of the literature. The search terms specifically related to tobacco interventions were informed by Almestahiri et al. [47], terms related to e-cigarette/vaping were informed by Calder et al. [48], terms related to heated tobacco products were informed by Jankowski et al. [49], and a consideration of social norms terms used in the tobacco literature was informed by East et al. [50].

### 2.3. Study Records

#### 2.3.1. Data Management

The complete search results will first be imported into Zotero to remove studies that have been retracted. They will then be imported on to the Covidence platform [51] for screening.

#### 2.3.2. Study Selection Process

At least two reviewers will independently screen studies following the revised PRISMA 2020 guidelines [52].The reviewers will initially screen titles and abstracts based on inclusion criteria, and will resolve discrepancies through discussion and consensus. The reviewers will then screen full-text articles following the same procedure to resolve discrepancies until a final list of included studies is determined. All reviewers will be public health doctoral students or candidates.

#### 2.3.3. Data Collection Process, Data Items, and Outcomes

Data from included studies will be extracted into an Excel spreadsheet, with rows representing individual studies and columns representing the extraction variables. A list of these variables along with descriptions and examples are provided in Table 1. These variables comprise typical study metadata such as author(s), year, population, and setting, as well as variables related to the four dimensions of effectiveness, measurement, modality, and mechanism.

The dimension of ‘effectiveness’ will be reflected in the meta-analyses, for which the sample size, effect size, and standard error for tobacco and social norms outcomes will be captured.

For the dimension of ‘measurement’, the focus will be on the items, scales, or indices used to measure social norms constructs (e.g., peer, injunctive, subjective), as well as the reference group used for social norms measurements. If the authors do not use terminology involving ‘norms’, information relating to their specific operationalization of social norms will be captured (e.g., social influence effects, perceived frequency/approval from others). If mentioned, the theory or framework used to inform their study will also be captured.

The dimension of ‘modality’ will be captured by information relating to the content, dose, and delivery of the intervention, as well as the role of social norms within it (if any). This is intended to understand the manner in which the intervention was delivered, specific social norms content (if any), and at what socioecological level the intervention was targeted (e.g., individual, interpersonal, community, society, multilevel). Relatedly, the total length of the intervention will also be captured to understand how effectiveness relates to the intervention dose delivered. (A more precise approach might be to capture average dose delivered to and received by the sample. Given that this review includes quasi-experimental studies as well, it is expected that this information will not be available in some studies in real-world settings (e.g., studies evaluating the impact of a law). Thus, length of the intervention was selected as a proxy.) The length of the intervention will be captured in the original metric provided in the study, even if it is a range.

Lastly, the dimension of ‘mechanism’ will be captured using the five categories from Legros & Cislaghi [43] noted in Section 1.2. Two additional categories will be created—a category when ‘Multiple’ mechanisms are used, as well as an ‘Other’ category to capture potential mechanisms that do not conform to the five categories. Relatedly, the social norms life stage—emergence, maintenance, and/or dissipation—mentioned by the authors will also be captured through a subjective assessment of the reviewers.

The main outcomes for this review that will be collected are those related to change in social norms and change in tobacco use. The primary analysis for this review will be conducted using peer-reviewed literature findings. A secondary analysis will be conducted using both the peer-reviewed and gray literature findings.

If data related to the social norms or tobacco outcomes are missing in any included studies, the original study authors will be contacted to see if they can provide the missing data. Study authors will also be contacted to retrieve full-text versions of included studies, if required.

#### 2.3.4. Risk of Bias in Individual Studies

Risk of bias assessment for individual studies will be conducted at the study level using the revised Cochrane Risk of Bias tool (ROB 2) [53] for randomized trials, and the ROBINS-I tool [54] for non-randomized studies. Results from risk of bias assessment will be reported in the study, and will be used to inform the interpretation of study findings.

### 2.4. Data

#### 2.4.1. Narrative Synthesis Approach

We will conduct a narrative synthesis of themes emerging from the studies to address the review questions, in line with guidance provided by [55]. Under this framework, the synthesis process is focused on addressing four key elements: (1) a theory for how the interventions work, and for whom; (2) developing a preliminary synthesis of included study findings; (3) exploring relationships in the data; and (4) assessing the robustness of the synthesis. The main narrative synthesis will be based on the peer-reviewed literature. This will be complemented by findings from the gray literature, highlighting the similarities and differences between gray literature and peer-reviewed literature.

#### 2.4.2. Meta-Analytic Approach

To address research question 1 about the effectiveness of social norms interventions, we will conduct a random effects meta-analysis of effect sizes. A random effects meta-analytic strategy is appropriate for this study as it assumes that there is no single true effect size (as a fixed effects approach assumes), but a distribution of true effect sizes. Given the varied intervention approaches, implementation strategies, target populations and settings to influence social norms around tobacco use, it is more reasonable to assume that there is a distribution of true effect sizes [56]. Conceptually, different social norms change mechanisms might have a distribution of true effect sizes for each mechanism, whereas the variation in the distribution may result from differences in intervention modality, target population, and context.

The estimate of the true effect size from a given study reflects the sum of a true effect and sampling error. This relationship is specified in Equation (1).

(1) $$\begin{array}{c} Y_{i}=\mu +\xi_{i}+\epsilon_{i} \end{array}$$

where the observed effect size of a given study $Y_{i}$ is the sum of the grand mean μ (mean effect size across all studies), the deviation between the observed effect and a true effect for a given study $\xi_{i}$, and the deviation between the study’s true effect and the grand mean $\epsilon_{i}$.

Additionally, μ depends on the variance of the distribution of true effect sizes $\tau^{2}$, and an estimate of $\tau^{2}$ is needed to compute μ. While the DerSimonian-Laird [57] method for calculating $\tau^{2}$ is a popular default estimator, recent evidence suggests that it has unfavorable statistical properties such as underestimating the true between-study variation [58]. While there are many estimators available, we will follow the recommendation of Veroniki et al. [58] to use a Restricted Maximum Likelihood estimator to calculate an estimate of $\tau^{2}$. Under this iterative approach, an estimate of $\tau^{2}$ is derived by setting the derivative of the restricted log-likelihood function with respect to $\tau^{2}$ to 0, and then solving the resulting equation [58,59].

Following this strategy, we will calculate effect size(s) for each study based on (a) intervention effects on tobacco use outcomes, and (b) intervention effects on social norms outcomes. For our primary analysis, we will conduct two meta-analyses—one for tobacco use outcomes, and another for social norms outcomes—based on peer-reviewed literature. Our secondary analysis will follow the same approach, but will incorporate grey literature findings as well. If a study has multiple effect sizes, perhaps even for different outcomes (e.g. e-cigarette use and cigarette use, or descriptive norms and injunctive norms), all relevant effect sizes will be extracted and included in the two meta-analyses. For studies that have an explicit social norms or social influence component in their intervention, but do not measure changes in social norms, only the tobacco use effect sizes will be used, due to unmeasured normative changes. The effect sizes will be a standardized mean difference (SMD), in the form of Hedges *g*. This summary measure is preferred to the more conventional Cohen’s *d* because the latter overestimates the SMD in small sample studies [60]. The calculation of Hedges *g* is shown in Equation (2):

(2) $$\begin{array}{c} g=\frac{M_{1}-M_{2}}{SDpooledandweighted}*\frac{N-3}{N-2.25}*\sqrt{\frac{N-2}{N}} \end{array}$$

where the mean difference between groups is divided by the pooled and weighted standard deviation, and multiplied by a correction factor.

A meta-analysis is expected to be possible given the results of a pilot search indicating sufficient quantitative studies. If however, the meta-analysis is not possible, the narrative summary of findings will suffice.

#### 2.4.3. Heterogeneity

To assess the magnitude of heterogeneity between studies, we will calculate the *Q* statistic, which is presented in Equation (3):

(3) $$\begin{array}{c} Q=\sum_{i=1}^{k}W_{i}{\left(Y_{i}-M\right)}^{2} \end{array}$$

where $W_{i}$ is the weight for a given study given by the inverse of the variance of the effect size, $Y_{i}$ is a study’s estimated effect size, *M* is the overall summary effect, and *k* is the number of studies. In this way, *Q* represents a weighted sum of squared deviations from the grand mean.

While this statistic is informative for understanding the magnitude of heterogeneity between studies, we will use it to calculate a commonly used and more intuitive measure of heterogeneity $I^{2}$, presented in Equation (4):

(4) $$\begin{array}{c} I^{2}=\frac{Q-df}{Q}*100\% \end{array}$$

where *df* represents the degrees of freedom (k−1) which is also the expected value of *Q*. Taking the difference between *Q* and *df* then provides an estimate of the excess variation attributed to differences in the true effects between studies. As a general guide, $I^{2}$ values of 25%, 50%, and 75% correspond to low, medium, and high heterogeneity respectively [61]. Thus, the $I^{2}$ statistic reflects the proportion of observed variance reflecting real differences in effect sizes. It can be considered a rudimentary signal-to-noise ratio of heterogeneity.

We will explore sources of heterogeneity using meta-regression with the effect size as the dependent variable, and one to two independent variables, depending on the number of retrieved studies containing the covariates. The following potential covariates are suggested a priori: social norms change mechanism, length of the intervention, whether the intervention is focused on prevention or cessation, and if the target population are adults vs non-adults. Not all social norms interventions are effective, and one potential reason may relate to the specific social norms change mechanism used. For instance, an individual-level mechanism may be used when a community-level mechanism may be more effective in particular settings. The length of the intervention was also considered to be highly relevant to intervention costing, which in turn are crucial for developing policies and programs. The focus of prevention or cessation are considered relevant as there are likely qualitative differences in interventions that prevent initiation of tobacco, compared with those focused on cessation among more experienced users. Finally, given the cognitive and developmental differences between adolescents and adults, we expect that whether the target population is under or over 18 is likely to contribute to heterogeneity between studies.

The social norms change mechanism covariate will be a nominal independent variable with five *a priori* categories corresponding to the five mechanisms noted by Legros & Cislaghi [43]. Two categories for ‘Other’ and ‘Multiple’ will be added to capture other mechanisms that do not conform to the other categories and situation where multiple mechanisms may be used. However, the levels may be collapsed into a smaller subset if there are limited studies at each covariate level. The length of intervention variable will initially be on the metric provided in the study. Prior to meta-regression, a standardized time metric will be created for this variable, taking the midpoint of any range provided. Thus, if a study provides the length of an intervention as “18–22 months”, this will be recorded as “20 months” using the the standardized time metric. This will create a continuous covariate for meta-regression. For policies or interventions that do not have an end date (e.g., cigarette taxes, smoking bans), the length of time will be based on the evaluation period noted in the study. The prevention/cessation covariate will comprise three categories, including one for ‘Both’. The target population covariate will follow the same coding scheme and will include three categories. As with the social norms mechanism covariate, the levels of each covariate may be collapsed if sufficient studies containing each covariate level are not retrieved.

The selection of additional covariates depends on the results of included studies. These might include type of tobacco product (e.g., ENDS/HTPs compared with traditional combustible products).

A prediction interval will be generated around the summary effect to determine how the true effects are distributed about the summary effect. If the prediction interval is narrow, for example, this suggests that the different social norms interventions have similar effects on tobacco use, and perhaps there is not much value added in more expensive interventions. By contrast, a wider interval will suggest greater variation in the effectiveness of each intervention approach, suggesting a greater value added for certain types of interventions.

### 2.5. Meta-Biases

Studies are more likely to be written up and published if they contain statistically significant results than if they do not contain statistically significant results [62]. To mitigate this type of non-reporting bias, We will include gray literature as part of the systematic search, and for sensitivity analyses. Secondly, we will also construct a contour-enhanced funnel plot, and will use Egger’s test to probe for asymmetry in the distribution of effect sizes.

Reporting for this review will follow the revised 2020 PRISMA Guidelines [52]. All meta-analytic procedures will be conducted in R [63] using the *metafor* package [64]. Forest plots will be generated for the social norms and tobacco use outcomes in order to visualize results. As with the narrative synthesis, the meta-analytic procedures will comprise a primary analysis using peer-reviewed literature, and a secondary analysis integrating gray literature as a sensitivity analysis.

### 2.6. Confidence in Cumulative Evidence

The strength of the overall quality of evidence will be discussed in the narrative by drawing upon the risk of bias findings.

## 3. Discussion and Limitations

As with any systematic review and meta-analysis, inferences arising from this paper are contingent on the quality of the included studies, and extent of publication bias in the literature. Though efforts to locate gray literature for the secondary analyses will be made in order to mitigate the influence of non-reporting biases, they will not capture studies which authors have relegated to the “file drawer” (i.e., not published) or those that have not been written up. Thus, the summary effects in the meta-analytic results may not represent the entire population of tobacco use interventions with social norms components. However, given the paucity of literature estimating the effect of such interventions in the tobacco literature, this paper will still provide a useful starting point for interventionists and researchers interested in understanding the role of social norms in tobacco use interventions. In particular, by delineating the effectiveness and approach to social norms measurement of included studies, this paper will be able to offer guidance as to the conditions under which such interventions are effective, as well as guidance for measurement best practices.

Given that the focus of this paper is on tobacco use interventions with explicit social norms components, it will necessarily exclude interventions that do not explicitly discuss social norms or social influence as part of the intervention or set of measured variables. Interventions without these components could still indirectly influence social norms, and these effects would not be captured in this paper. However, without measuring social norms in some fashion, it is not possible to quantify or understand their influence whether as a direct or indirect outcome. Thus, while this study focuses on the role of social norms in tobacco interventions, it will not be representative of all tobacco use interventions that can influence social norms.

## 4. Conclusions

This systematic review and meta-analysis will broaden the existing knowledge base for social norms interventions. Our hope is that our approach to defining a “social norms intervention” captures the diversity inherent in such interventions, and provides a useful perspective for understanding how social norms can manifest and be shifted at multiple socioecological levels. By focusing on the underlying mechanisms of change, our approach is intended to capture the active ingredients of such interventions, to facilitate generalization to other behaviors. This will allow program planners and policy makers to consider the conditions under which particular *mechanisms*, rather than just intervention modalities, are most and least effective, prior to investing resources in norms-shifting efforts. This study was pre-registered on the PROSPERO database of systematic reviews on 27 May 2021 [CRD42021251535].

## Figures and Tables

**Table 1:** The list of variables to be extracted and their descriptions.

Extraction Variable	Description
Article Information	Author(s), year, country(ies), population(s).
Study Design	Specific type of randomized or non-randomized trial.
Sample size	Final sample used to estimate tobacco outcomes and social norms outcomes (if applicable).
Effect size(s) and standard error(s)	Effect size(s) converted to Hedges *g* with standard error(s) for tobacco outcome and social norms outcome (if applicable).
Social norms conceptualization	Theoretical approach mentioned (e.g., Theory of Normative Social Behavior, Theory of Planned Behavior, etc), if any.
Social norms measurement approach	How norms were measured in the study (e.g., descriptive norms, injunctive norms, etc) including a description of the items, scales, or indices used to measure them, along with an indication of internal consistency (e.g., Cronbach’s alpha), if any.
Social norms reference group measurement approach	What reference group(s) was/were used for social norms measures (e.g., peers, society at large, etc). If no reference group is provided, and the reference group cannot be discerned by the social norms measurement approach, this will be assigned a value of “NONE”.
Intervention modality and role of social norms	The form that the intervention took in the study. For instance, a radio soap opera is a modality, as is a poster presenting normative information. Multicomponent and multilevel intervention details will also be captured. Additionally, a brief description of how the intervention targeted or incorporated social norms, including social influence channels (e.g., using peers as social influence levers of smoking cessation). If normative information was presented, how was this information framed?
Length of the intervention	How long the intervention was run for among the treatment group. Ranges are acceptable (midpoint will be taken later). Length of intervention should be in the metric provided in the study.
Social norms change mechanism	Underlying active ingredient(s) of social norms change approach (e.g., correcting misperceptions).
Social norms life stage targeted by intervention	Indication of whether intervention is conducted at emergence, maintenance, or dissipation stage of social norms life cycle.
Prevention or cessation	Based on what is identified by the authors, this will capture whether the intervention is intended to prevent initiation of tobacco, or cessation among existing users, or both.
Adults or non-adults	Based on what is identified by the authors, this will capture whether the intervention is targeted at adults, non-adults, or both. The age of adulthood will be determined by the jurisdiction and time in which the study took place.

## Supplementary Materials

The following are available online at https://www.mdpi.com/article/10.3390/ijerph182212186/s1, PRISMA-P Checklist (S1) and full search strategy for PubMed (S2).
