# Supplementary material for: Social Norms Change and Tobacco Use: A Protocol for a Systematic Review and Meta-Analysis of Interventions
Source: Int J Environ Res Public Health. 2021 Nov 20;18(22):12186. doi: 10.3390/ijerph182212186 (PMC8618876; doi:10.3390/ijerph182212186)
Supplement: Supplementary file 1 [file ijerph-18-12186-s001.zip › Supplementary File S2.pdf]

Supplementary File S2 – Complete Search for PubMed

First, I used the following term groups to create five separate entries.

1. cig* OR tobacco OR smok* OR dip OR water pipe OR hookah OR shisha OR bidi OR tobacco prevalence OR smoking prevalence OR ends OR vap* OR heat-not-burn OR iqos

2. norm OR social influenc* OR peer influenc* OR famil* influenc* OR friend influenc* OR approv*

3. random* OR trial OR experiment OR "nonequivalent groups" OR "cohort study" OR longitudinal OR prospective OR retrospective OR "propensity score matching" OR "regression discontinuity" OR "difference-in-difference" OR "double difference" OR "instrumental variables" OR "time series" OR case-control OR quasi-experiment* OR pre-post OR case-cohort OR evaluation

4. mhealth OR digital OR "text messag* " OR nrt OR medic* OR media OR campaign* OR policy OR tax* OR law OR intervention OR counsel* OR peer OR group OR sms OR game* OR monetary OR incentiv* OR educat*

5. "systematic review" OR "scoping review" OR "rapid review" OR "cross-sectional" OR protocol

I then combined them all using Boolean operators to yield the complete search for PubMed (next page):

(((((((((norm[Title/Abstract]) OR (social influenc*[Title/Abstract])) OR (peer influenc*[Title/Abstract])) OR (famil* influenc*[Title/Abstract])) OR (friend influenc*[Title/Abstract])) OR (approv*[Title/Abstract])) AND ((((((((((((((cig*[Title/Abstract]) OR (tobacco[Title/Abstract])) OR (smok*[Title/Abstract])) OR (dip[Title/Abstract])) OR (water pipe[Title/Abstract])) OR (hookah[Title/Abstract])) OR (shisha[Title/Abstract])) OR (bidi[Title/Abstract])) OR (tobacco prevalence[Title/Abstract])) OR (smoking prevalence[Title/Abstract])) OR (ENDS[Title/Abstract])) OR (vap*[Title/Abstract])) OR (heat-not-burn[Title/Abstract])) OR (iqos[Title/Abstract]))) AND (((((((((((((((((((random*[Title/Abstract]) OR (trial[Title/Abstract])) OR (experiment[Title/Abstract])) OR (nonequivalent groups[Title/Abstract])) OR (cohort study[Title/Abstract])) OR (longitudinal[Title/Abstract])) OR (prospective[Title/Abstract])) OR (retrospective[Title/Abstract])) OR (propensity score matching[Title/Abstract])) OR (regression discontinuity[Title/Abstract])) OR (difference-in-difference[Title/Abstract])) OR (double difference[Title/Abstract])) OR (instrumental variables[Title/Abstract])) OR (time series[Title/Abstract])) OR (case-control[Title/Abstract])) OR (quasi-experiment*[Title/Abstract])) OR (pre-post[Title/Abstract])) OR (case-cohort[Title/Abstract])) OR (evaluation[Title/Abstract]))) AND (((((((((((((((((((mhealth[Title/Abstract]) OR (digital[Title/Abstract])) OR (text messag*[Title/Abstract])) OR (NRT[Title/Abstract])) OR (medic*[Title/Abstract])) OR (media[Title/Abstract])) OR (campaign*[Title/Abstract])) OR (policy[Title/Abstract])) OR (tax*[Title/Abstract])) OR (law[Title/Abstract])) OR (intervention[Title/Abstract])) OR (counsel*[Title/Abstract])) OR (peer[Title/Abstract])) OR (group[Title/Abstract])) OR (sms[Title/Abstract])) OR (game*[Title/Abstract])) OR (monetary[Title/Abstract])) OR (incentiv*[Title/Abstract])) OR (educat*[Title/Abstract]))) NOT (((((systematic review[Title/Abstract]) OR (scoping review[Title/Abstract])) OR (rapid review[Title/Abstract])) OR (cross-sectional[Title/Abstract])) OR (protocol[Title/Abstract]))
